# The Treatment of Lunatics

**Published:** 1916-12-02

**Authors:** 


### The Hospital

The Workers' Newspaper of Administrative Medicine and Institutional Life, Administration, National Insurance and Health.

No. 1591, Vol. LXI. SATURDAY, DECEMBER 2, 1916. PRICE ONE PENNY.

### THE TREATMENT OF LUNATICS.

Medical practitioners as a rule know little of the law of lunacy and nothing at all of mental disease. The field of medicine is now so vast that it is not within human capacity to master the whole of it, and the specialty of mental disease is one of the oldest and most isolated specialties. It is not to be expected that the general practitioner, still less that the expert in other departments of medicine, should have any more than the most general knowledge of mental disease, but they ought to know enough to be able to recognise it when it exists, to know when the services of a specialist are required, and not to go on attending a case of established insanity in the belief that it is "only" a case of "nervous breakdown," or hysteria, or neurasthenia, or "nerves," or "temper." Even this degree of knowledge is not common. It is frequently wanting, not only in the general practitioner, but also in the neurologist, to whom his reputation as "a great authority on the brain" usually attracts cases of mental disease in the first instance. The difference between diseases of the nervous system, evidenced in paralysis or spasm or pain, and diseases of the mind, evidenced in defect or disorder of conduct, vast and profound as the difference is, is never appreciated by the laity, and insufficiently by the medical profession. Even a surgeon who has attained skill and experience in operating on the skull and the brain has attracted to himself, as "a great authority on the brain," a very large practice in mental diseases, a result about as rational as consulting a builder on difficult problems of history, on the ground that he has repaired the library of the Historical Society. It is possible, of course, that a neurologist may know something of mental disease; but if he does, it is not because he is a neurologist, but because, in addition to neurology, he has studied another specialty. It is possible, too, that a surgeon may know something of mental disease; but if he does, it is not because he is a surgeon, but because he has been attracted to study a subject outside the realm of surgery. But as a rule neither the surgeon nor the neurologist who dabbles in mental diseases has enough knowledge of them even to suspect how ignorant he is; and as a rule his treatment of them is the merest routine, and is about as intelligent a response as might be obtained from putting a guinea in a slot and drawing out a prescription. It consists in sending the patient, according to the habit of the consultant, either for a sea voyage, or to travel, or to a farmhouse, or to a nursing home, where he is usually massaged and fed upon milk.

Each of these routine methods has its special consequences. The sea voyage is commonly advised for patients who are depressed, melancholy, and weary of life, and the conditions of a sea voyage are such as to offer no distraction to their melancholy imaginings, to add the weariness of monotony, lack of occupation, and lack of interest to their already wearied minds; and to offer to them the ever-present opportunity and temptation to suicide. The consequence is such as may be expected, but for obvious reasons precautions are taken against their attaining publicity, and no one but the officers at headquarters of the great steamship lines has any idea of the number of suicides that are annually effected in this manner. The farmhouse treatment, the travelling, and the nursing home with the massage and milk, each has its own special disadvantages and its own special danger, as inquests from time to time remind us; and all have in common the uniform disadvantage that the patient is worse at the end of the treatment than he was at the beginning.

From the point of view of the farmer, and of the matron or proprietor of the nursing home, another defect of these methods is that they are violations of the law, and expose those who take part in them to prosecution for misdemeanour. The farmer or the matron does not know this. Usually they have no suspicion of it, for they know nothing of lunacy law; and if they have any suspicion, their uneasiness is laid at rest, partly by the misleading appellation—nerves, or nervous breakdown, or hysteria, or neurasthenia—that is given to the case, and partly by their confidence in the "eminent specialist" by whom the case is sent to them. There are in and near London several nursing homes that habitually "for payment take charge of, receive to board and lodge," and more or less actively "detain" lunatics who may or may not be suspected to be lunatics, but whose lunacy is called by another name, and therefore, it is thought, may successfully, and perhaps even truthfully, be denied. Such a course is full of peril, both for the patient and for the matron or proprietor of the home. For the patient because the nature of his disease is misconceived and misunderstood, and the treatment he receives is inappropriate and probably damaging; for the matron or proprietor of the home, because she is running a risk of prosecution. She is apt to suppose that the responsibility lies on the eminent specialist who has placed the patient with her, and that if she carries out his orders she is safe. This is quite a mistake. No doubt he incurs a very serious moral responsibility, but he incurs no legal responsibility, and if the matter is taken up by the proper authorities, it is she and not he that will suffer.

